# Supplementary material for: Geranium and Lemon Essential Oils and Their Active Compounds Downregulate Angiotensin-Converting Enzyme 2 (ACE2), a SARS-CoV-2 Spike Receptor-Binding Domain, in Epithelial Cells
Source: Plants (Basel). 2020 Jun 19;9(6):770. doi: 10.3390/plants9060770 (PMC7355681; doi:10.3390/plants9060770)
Supplement: Supplementary file 1 [file plants-09-00770-s001.pdf]

Supplementary information

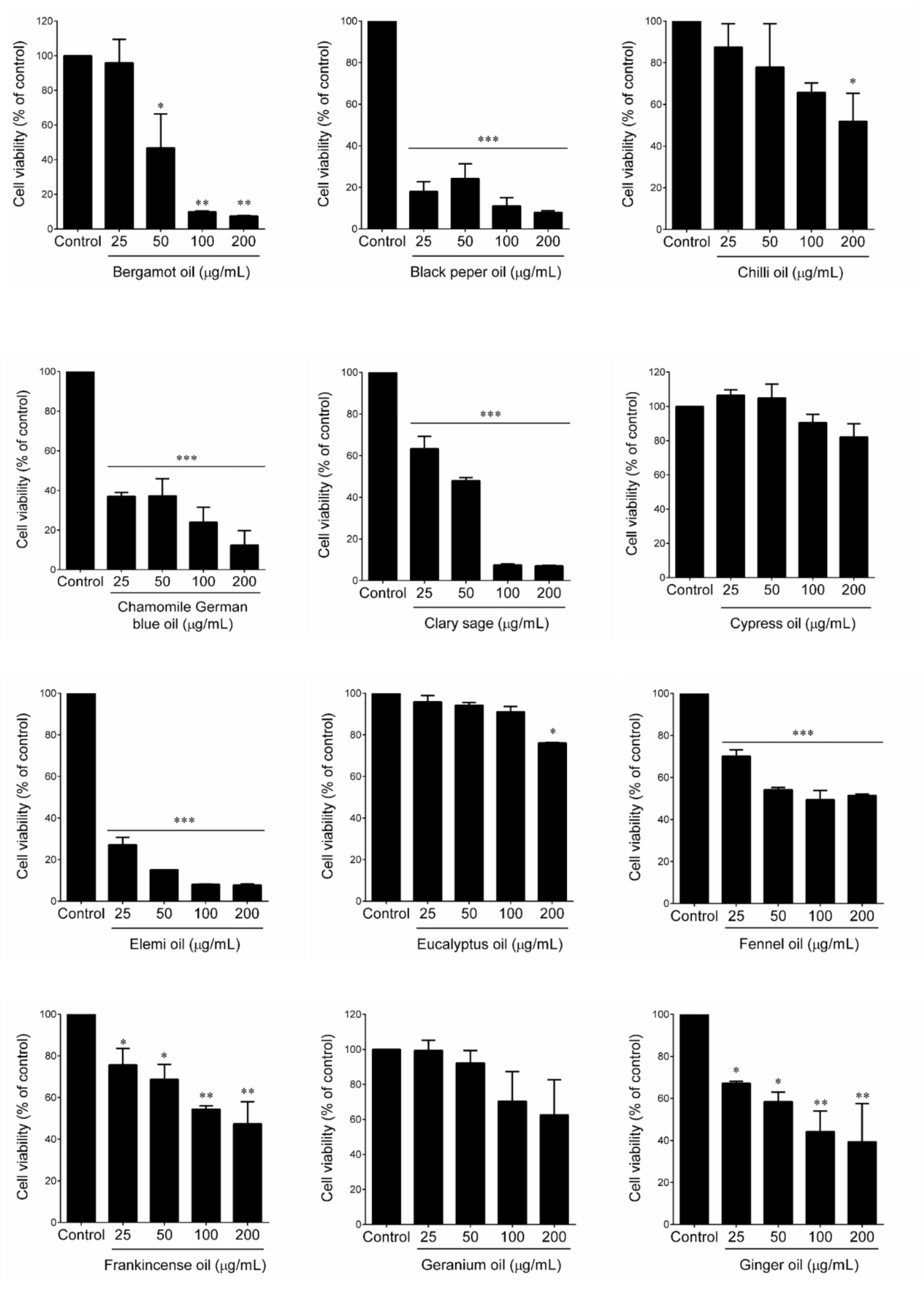

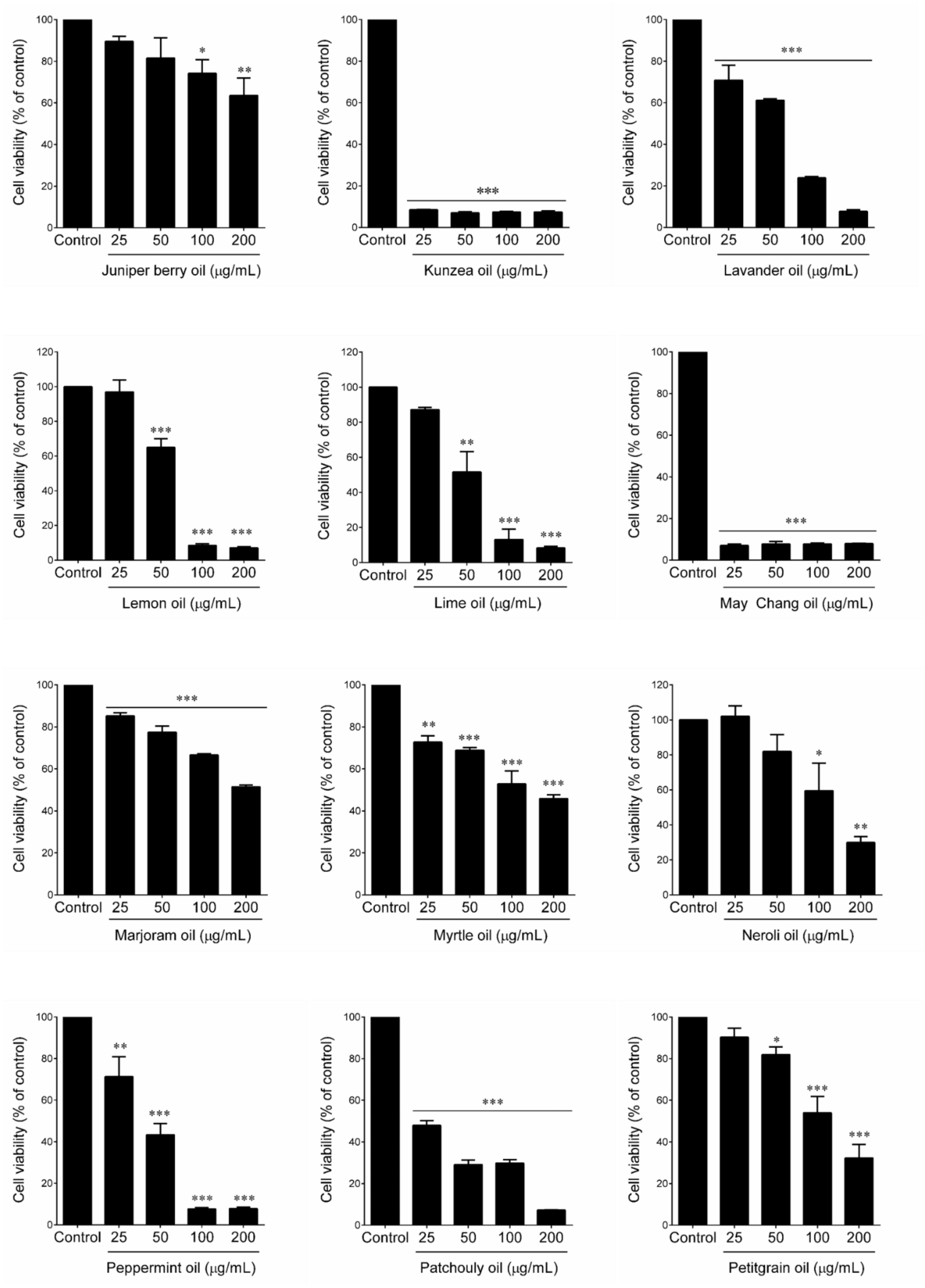
Cell viability (% of control)
Control 25 50 100 200
Juniper berry oil (μg/mL)
Kunzea oil (μg/mL)
Lavander oil (μg/mL)
Lemon oil (μg/mL)
Lime oil (μg/mL)
May Chang oil (μg/mL)
Marjoram oil (μg/mL)
Myrtle oil (μg/mL)
Neroli oil (μg/mL)
Peppermint oil (μg/mL)
Patchouly oil (μg/mL)
Petitgrain oil (μg/mL)

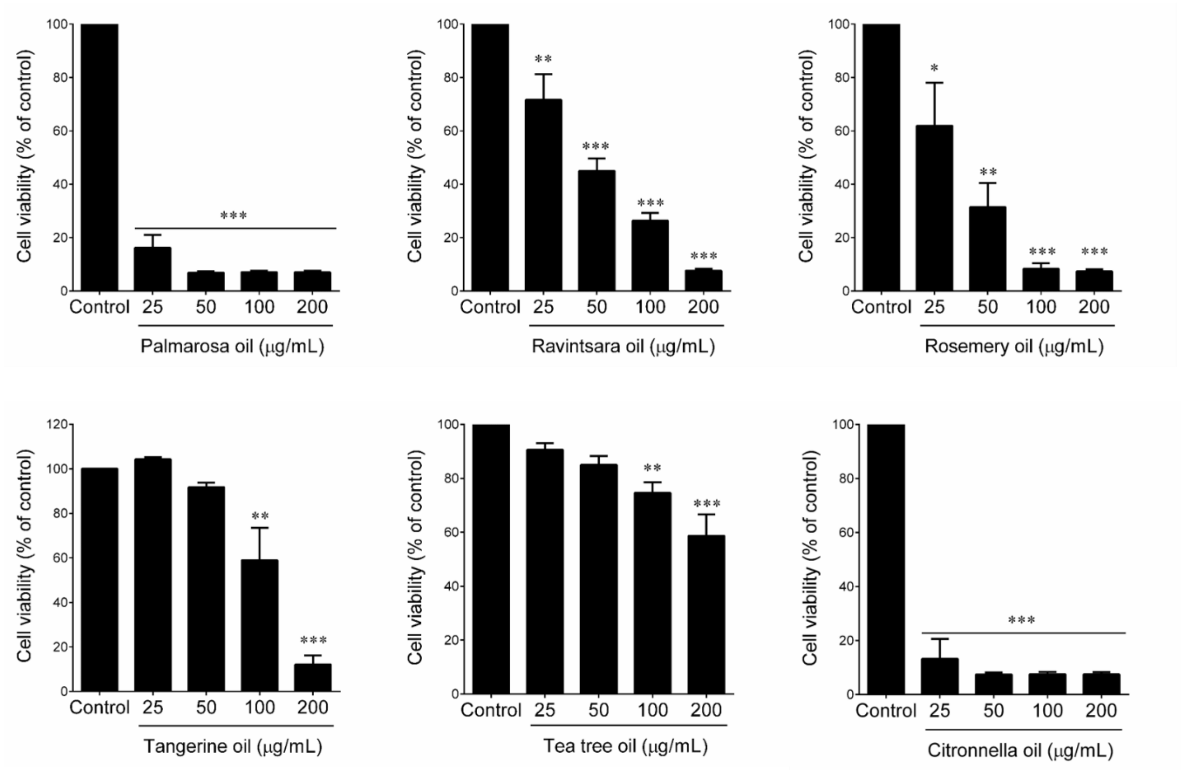

Figure S1. Cytotoxic effects of essential oils on HT-29 cells. HT-29 cells were incubated with increasing concentrations of essential oils (25-200 μg/mL) for 48 h. The cell viability was determined by the MTT colorimetric assay as described in materials and methods. Values represent the mean ± SD of three independent experiments. *P* values of less than 0.05*, 0.01**, and 0.001*** were considered statistically significant for the sample treatment group *vs.* the control group.

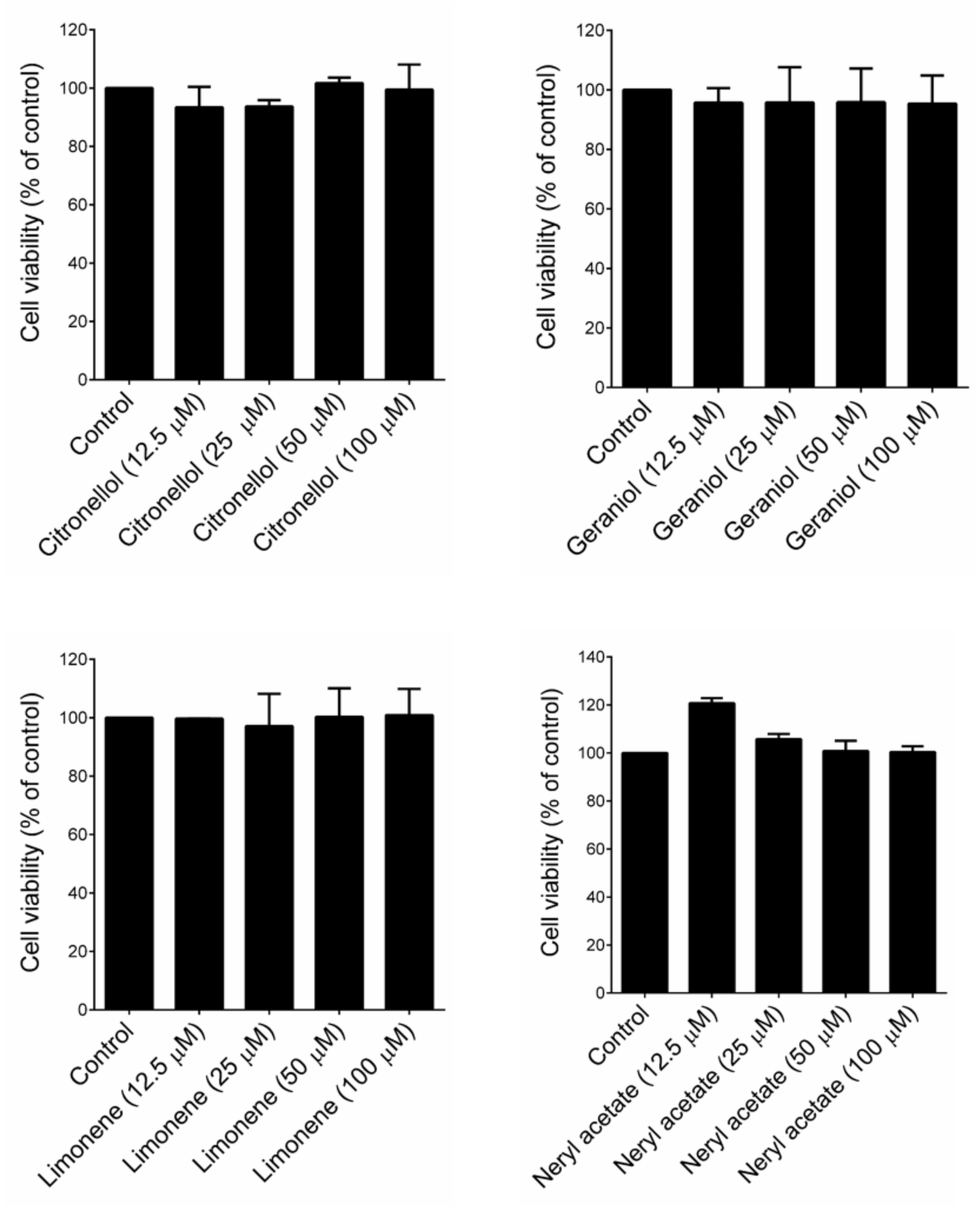

Figure S2. Cytotoxic effects of major compounds in geranium and lemon essential oils on HT-29 cells. HT-29 cells were incubated with increasing concentrations of pure compounds (12.5-100 µM) for 48 h. The cell viability was determined by the MTT colorimetric assay as described in materials and methods. Values represent the mean ± SD of three independent experiments.

Table S1. Main components and their relative contents (%) of Bergamot oil derived from citrus rind (peel) of *Citrus bergamia*.

S.No	RT (min)	Compound	Contents (%)	KI
1	10.28	α-Pinene	0.83	934.1944
2	12.06	Sabinene	0.48	973.2925
3	12.26	β-Pinene	3.71	977.3195
4	12.89	β-Myrcene	0.33	989.588
5	14.49	*p*-Cymene	2.64	1024.405
6	14.71	Limonene	37.65	1029.342
7	16.12	γ-Terpinene	5.5	1059.335
8	17.41	Terpinolene	0.16	1084.56
9	18.18	Linalool	10.15	1098.741
10	22.63	α-Terpineol	0.19	1192.815
11	24.64	Neral	0.1	1237.704
12	25.26	Linalyl acetate	36.82	1251.394
13	26	Geranial	0.19	1267.301
14	30.08	Neryl acetate	0.31	1358.893
15	32.58	β-Caryophyllene	0.15	1416.099

KI: Kovats index on a DB-5MS column in reference to *n*-alkanes. RT: Retention time.

Table S2. Main components and their relative contents (%) of cypress oil derived from needles of *Cupressus sempervirens*.

S.No	R.T (min)	Compounds	Contents (%)	KI
1	10.25	α-Pinene	50.1	**933**
2	10.95	Camphene	0.4	**950**
3	12.22	β-Pinene	1.9	**977**
4	12.86	β-Myrcene	0.7	**989**
5	13.69	3-Carene	20.8	**1006**
6	14.45	*p*-Cymene	1.3	**1023**
7	14.67	Limonene	4.2	**1028**
8	16.07	γ-Terpinene	0.5	**1058**
9	17.37	Terpinolene	1.7	**1084**
10	21.88	Terpinen-4-ol	0.7	**1178**
11	22.58	α-Terpineol	0.2	**1192**
12	26.69	L-bornyl acetate	0.6	**1282**
13	29.45	Nerol acetate	3.0	**1344**
14	32.06	Longifolene	0.3	**1403**
15	32.35	α-Cedrene	1.2	**1410**
16	32.51	β-Caryophyllen	0.5	**1414**
17	32.67	β-Cedrene	0.3	**1418**
18	33.14	Thujopsene	0.3	**1430**
19	33.99	α-Humulene	0.1	**1451**
20	34.82	γ-Muurolene	0.3	**1471**
21	35.04	Germacrene D	0.1	**1476**
22	35.8	α-Muurolene	0.2	**1494**
23	36.59	γ-Cadinene	0.9	**1514**
24	37.44	α-Calacorene	0.1	**1536**
25	39.01	Caryophyllene oxide	0.1	**1576**
26	40	Cedrol	6.5	**1601**

KI: Kovats index on a DB-5MS column in reference to *n*-alkanes. RT: Retention time.

Table S3. Main components and their relative contents (%) of Eucalyptus oil derived from leaves of *Eucalyptus globulus*.

S.No	RT (min)	Compound	Contents (%)	KI
1	14.84	Eucalyptol	82.9	**1032**
2	10.30	α-Pinene	2.5	**935**
3	12.26	β-Pinene	0.2	**977**
4	12.91	β-Myrcene	0.2	**990**
5	13.62	α-Phellandrene	0.6	**1004**
6	14.49	*p*-Cymene	3.7	**1024**
7	14.72	Limonene	4.4	**1030**
8	16.12	γ-Terpinene	5.0	**1059**
9	17.41	Terpinolene	0.1	**1085**

KI: Kovats index on a DB-5MS column in reference to *n*-alkanes. RT: Retention time.

Table S4. Main components and their relative contents (%) of juniper berry oil derived from berries of *Juniperus communis.*

S.No	RT (min)	Compound	Contents (%)	KI
1	10.32	α-Pinene	57.95	**935**
2	11.03	Camphene	0.89	**951**
3	12.10	Sabinene	0.27	**974**
4	12.30	β-Pinene	10.99	**978**
5	12.94	β-Myrcene	1.72	**991**
6	13.76	3-Carene	7.63	**1007**
7	14.52	*p*-Cymene	1.70	**1025**
8	14.75	Limonene	6.05	**1030**
9	17.44	Terpinolene	0.31	**1085**
10	22.66	α-Terpineol	0.58	**1193**
11	26.77	Bornyl acetate	1.80	**1283**
12	29.57	α-Cubebene	0.17	**1347**
13	30.76	α-Copaene	0.24	**1374**
14	32.14	Longifolene	2.64	**1405**
15	32.60	β-Caryophyllene	3.96	**1417**
16	34.07	α-Humulene	0.39	**1453**
17	36.66	δ-Cadinene	0.50	**1516**
18	39.09	Caryophyllene oxide	0.98	**1578**

KI: Kovats index on a DB-5MS column in reference to *n*-alkanes. RT: Retention time.

Table S5. Main components and their relative contents (%) of neroli oil derived from flowers of *Citrus aurantium*.

S.No	R.T (min)	Compound	Contents (%)	KI
1	14.71	Limonene	12.0	1029
2	18.17	Linalool	14.6	1099
3	18.62	Phenylethyl Alcohol	4.4	1109
4	22.62	α-Terpineol	1.1	1193
5	25.23	Linalyl acetate	33.3	1251
6	29.09	Methyl anthranilate	9.4	1336
7	29.49	δ-Elemene	1.2	1345
8	29.56	α-Terpinyl acetate	0.4	1347
9	30.06	Neryl acetate	7.6	1358
10	30.92	Geranyl acetate	10.6	1378
11	32.55	β-Caryophyllene	0.2	1415

KI: Kovats index on a DB-5MS column in reference to *n*-alkanes. RT: Retention time.

Table S6. Main components and their relative contents (%) of petitgrain oil derived from leaves and twigs of *Citrus aurantium*.

S.No	RT (min)	Compound	**Contents (%)**	KI
1	12.29	β-Pinene	0.5	978
2	12.93	β-Myrcene	0.7	990
3	13.76	3-Carene	0.2	1007
4	14.74	Limonene	0.6	1030
5	15.13	β-Ocimene	0.4	1039
6	15.63	*trans*-β-Ocimene	1.1	1049
7	17.44	Terpinolene	0.3	1085
8	18.19	Linalool	18.6	1099
9	22.65	α-Terpineol	6.9	1193
10	24.06	Nerol	0.3	1225
11	25.27	Linalyl acetate	62.1	1252
12	29.52	α-Terpinyl acetate	0.1	1346
13	30.09	Neryl acetate	2.5	1359
14	30.94	Geranyl acetate	4.0	1378
15	32.59	Caryophyllene	1.1	1416
16	34.07	α-Humulene	0.1	1453

KI: Kovats index on a DB-5MS column in reference to *n*-alkanes. RT: Retention time.

Table S7. Main components and their relative contents (%) of tangerine oil derived from fruit peel of *Citrus reticulata*.

S.No	RT (min)	Compound	Contents (%)	KI
1	10.26	α-Pinene	0.5	934
2	12.04	β-Pinene	0.1	973
3	12.86	β-Myrcene	0.7	989
4	14.45	*p*-Cymene	0.6	1023
5	14.68	Limonene	96.5	1029
6	16.08	γ-Terpinene	1.2	1059
7	18.14	Linalool	0.1	1098
8	35.51	(+)-Valencene	0.1	1487

KI: Kovats index on a DB-5MS column in reference to *n*-alkanes. RT: Retention time.

Table S8. Main components and their relative contents (%) of tea tree oil derived from leaves of *Mellaleuca alternifolia*.

S.No	RT (min)	Compound	Contents (%)	KI
1	14.16	α-Terpinene	0.1	1017
2	14.51	*p*-Cymene	0.2	1025
3	14.80	β-Ocimene	0.1	1031
4	16.15	γ-Terpinene	0.1	1060
5	17.43	Terpinolene	0.2	1085
6	17.66	*p*-Cymenene	0.0	1089
7	18.18	Linalool	0.1	1099
8	19.30	Allo-Ocimene	0.4	1124
9	21.97	Terpinen-4-ol	97.9	1180
10	25.35	β-Citral	0.3	1253

KI: Kovats index on a DB-5MS column in reference to *n*-alkanes. RT: Retention time.
